# Supplementary material for: Personalized immunoglobulin aptamers for detection of multiple myeloma minimal residual disease in serum
Source: Commun Biol. 2020 Dec 17;3:781. doi: 10.1038/s42003-020-01515-x (PMC7747622; doi:10.1038/s42003-020-01515-x)
Supplement: Supplementary file 2 — Supplementary Information [file 42003_2020_1515_MOESM2_ESM.pdf]

Supplementary methods and results

Personalized immunoglobulin aptamers for detection of multiple myeloma minimal residual disease in serum

Short title

Fab aptamers for MM MRD serum monitoring

Claudia Tapia-Alveal, Timothy R. Olsen, Tilla S. Worgall*
Department of Pathology and Cell Biology, Columbia University Irving Medical Center, New York, New York 10032, USA

Correspondence

*Tilla S. Worgall
Dept. of Pathology and Cell Biology
Columbia University Medical Center
630 West 168th Street
P & S Building 15-409
New York, NY 10032
(212) 305-2498
tpw7@cumc.columbia.edu

Supplementary methods

M-Ig purification. One to five ml of MM patient serum (M-Ig >1 g/dl) were subjected to Melon gel IgG columns (Thermo Scientific cat # 45206) following manufacturer instructions. For SPR assays, IgGs were additionally purified on protein G columns (Santa Cruz Biotechnology sc-2002). Immunoglobulins were washed with PBS, and eluted with IgG elution buffer (Thermo Scientific cat # 21004). Purified M-Ig yields were measured by optical density at 280 nm and purity was verified using tris-glycine gels (Invitrogen Thermo Scientific, Novex WedgeWell cat # XP00100BOX and cat # XP10200BOX). SYPRO Ruby Protein Gel Stain (Thermo Scientific cat # S12000) was used to visualize the bands on a standard UV transilluminator.

FragIT (IdeS, GENOVIS A2-FR2-005) and FabALACTICA (IgdE, GENOVIS A2-AFK-005) digestion. Daratumumab (0.3 mg) was digested following the manufacturer's 'microspin' protocol. For FabALACTICA digestion, daratumumab was buffer exchanged to digestion buffer (0.2 M sodium phosphate, pH 7.2, Alfa Aesar, cat # J63816). Fragments were collected in the flow-through according to the manufacturer instructions. CaptureSelect Fc resin was used for affinity binding of the Fc fragments, eluted with IgG elution buffer, and exchanged to SELEX buffer using Zeba columns (7K MWCO, 0.5 mL, Thermo Scientific, cat # 89882). Collections were analyzed by SDS-PAGE and ELONA [38,39].

Supplementary Figure 1

Supplementary results

a

**Stages**	**Procedure**
ssDNA library preparation	1 nmol of ssDNA is used for round 1 and 80 pmols for subsequent rounds. Final volume is 200 µl (SB). 30 minutes incubation with selection target. SELEX Buffer (SB): PBS, 2 mM $MgCl_{2.}$
Selection	3 nmol of target antibody immobilized to protein G beads (200 µl of 1:1 slurry of protein G beads previously washed with SB, efficient immobilization can be monitored by saving aliquots for SDS PAGE analysis) are incubated for 30 minutes at room temperature with ssDNA. Washes are performed, followed by elutions (see below). Elutions are concentrated to 70 µl and washed with Molecular Biology grade water.
Counter-selection A	3 nmol of polyclonal IgG are immobilized to 200 µl of 1:1 slurry of protein G beads previously washed with SB. ssDNA eluted from the selection step is incubated for 30 minutes with the counter-target. Flow through is collected. ssDNA bound to poly-IgG column is discarded.
Counter-selection B	200 µl of empty protein G beads (1:1) washed in SB. Flow-through coming from counter-selection A is collected and incubated for 30 minutes with empty protein G beads. Flow through is collected and ssDNA bound to protein G beads is discarded.
Washes	5x with 800 µl of SB.
Elution	3x 10 minutes incubation with 400 µl of PBS, 2 mM EDTA. Elutions are pooled (recovered sequences) and concentrated to 70 µl on 10K MWCO Amicon Ultra centrifugal filter and washed with water 2x.
PCR	Fwd:5' GGAGGCTCTCGGGACGAC3' Rev:5'CTCAACTGGTCAGCAGCGAT3' Biotinylated Rev: 5' Biosg CTCAACTGGTCAGCAGCGAT3'. Annealing temperature is 60°C. Biotinylated PCR product is cleared from PCR reagents and concentrated on 10K MWCO Amicon Ultra centrifugal filter to 200 µl in PBS.
ssDNA recovery	Biotin labeled PCR amplicons are incubated 10 minutes with streptavidin agarose beads (200 µl 1:1 slurry previously washed 4x with 400 µl of PBS); washed 3x with 800 µl of PBS; ssDNA is recovered after 10 minutes incubation in 250 µl of 0.2 N NaOH; flow through is collected and neutralized with 0.2 N HCl. The pH and ionic conditions are adjusted by adding 10x PBS. The recovered ssDNA concentration is calculated to be used in the next round.

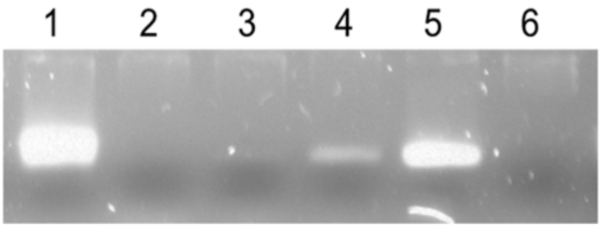

1= Flow through library
2= Wash #5
3= Elution 2
4= Elution 3
5= PCR positive control
6= PCR negative control

b

c

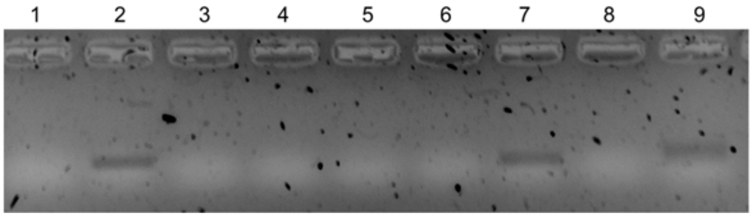

Lane	Condition	# of cycles
1=	Negative ctrl.	8
2=	Positive ctrl.	8
3=	Pooled ssDNA	10
4=	Negative ctrl.	12
5=	Pooled ssDNA	12
6=	Negative ctrl.	14
7=	Pooled ssDNA	14
8=	Negative ctrl.	16
9=	Pooled ssDNA	16

d

PCR cycles used to amplify recovered ssDNA in Daratumumab SELEX:

SELEX round	# of PCR cycles
1	13
2	14
3	15
4	13
5	9
6	7

e

Distribution of isolated sequences with Daratumumab (6 SELEX rounds; 20 sequences)
38% were aptD or related (fragments) that bound to daratumumab in buffer and in spiked serum. 62% were a second aptamer that bound to daratumumab in buffer and in serum that is not shown in here, as it is used for a different application.

SELEX procedure outline. (**a**) SELEX round stages. (**b**) Example of evaluation of ssDNA elutions aliquots. 3% agarose gel visualized with EtBr. (**c**) An aliquot of pooled and concentrated eluted ssDNA is tested for PCR amplification. Aliquots are loaded on 3% agarose gel and visualized with EtBr. Inverted digital image. After 14 PCR cycles (7) there is optimal amplification. At 16 PCR cycles (9) the band is shifted up in comparison with the positive control (2). (**d**) The progress of SELEX is evaluated by comparing the cycles of PCR required to amplify the pooled eluted ssDNA. Here, after round 4 of SELEX there is a significant drop in the amount of PCR cycles, indicating enrichment of sequences. (**e**) Two sequences were isolated from the Daratumumab SELEX.

Supplementary Figure 2

a

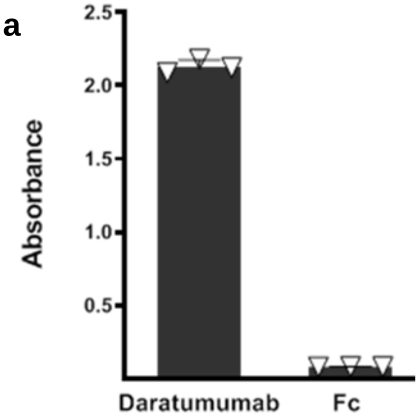

b

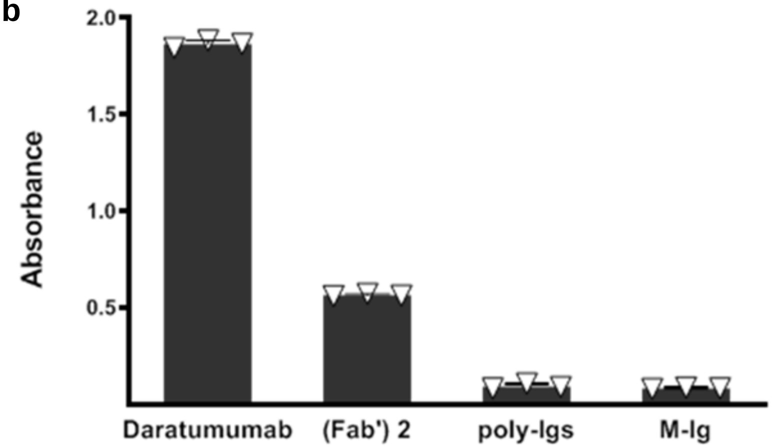

AptD binding to daratumumab Fc and (Fab')$_2$ fragments. (**a**) Daratumumab and daratumumab Fc fragment (1 pmol) were bound to protein G plates and incubated with aptD. AptD does not bind to the Fc fragment of daratumumab. (**b**) Biotin-aptD was immobilized on streptavidin coated plates and incubated with 20 pmols of daratumumab (Fab')$_2$ or control IgGs (daratumumab, polyclonal immunoglobulins, M-Igs). AptD binds to daratumumab and daratumumab (Fab')$_2$ fragment, but not to polyclonal immunoglobulins (poly-Igs) or M-Igs. Binding was detected with streptavidin-HRP with TMB (370 nm) after 20 minutes. All data points represent means plus standard deviations (n=3 biologically independent samples).

Supplementary Figure 3

a

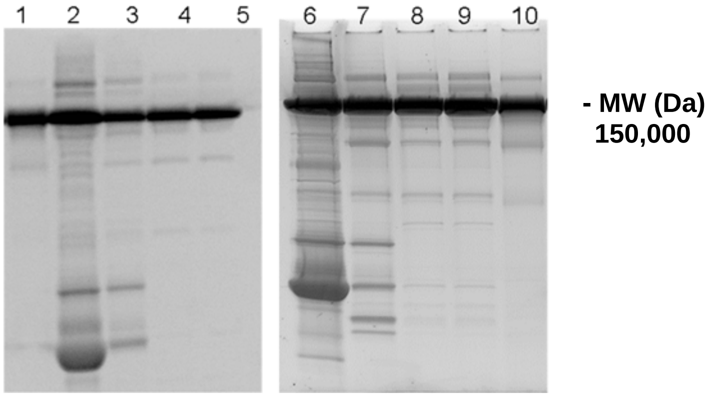

b

PCR cycles used to amplify recovered ssDNA in SD M-Ig SELEX:

SELEX round	# of PCR cycles
1	17
2	21
3	18
4	14
5	10
6	11
7	8

c

PCR cycles used to amplify recovered ssDNA in CR M-Ig SELEX:

SELEX round	# of PCR cycles
1	14
2	18
3	16
4	18
5	16
6	9

d

Distribution of isolated sequences with SD M-Ig (7 SELEX rounds; 19 sequences)
37% were aptS and bound to purified SD M-Ig in buffer and in serum
21% bound to purified SD M-Ig but did not bind in serum
42% were single sequences and did not bind to purified SD in buffer or serum

e

Distribution of isolated sequences with CR M-Ig (6 SELEX rounds; 19 sequences)
10% were aptC and bound to purified CR M-Ig in buffer and serum
50% (same sequence) bound purified CR M-Ig in buffer but did not bind in serum
20% (same sequence) did not bind to purified CR in buffer or serum
10% (same sequence) did not bind to purified CR in buffer or serum
10% (same sequence) did not bind to purified CR in buffer or serum

Patient M-Ig purification and SELEX stages. (**a**) Serum samples were subjected to Melon gel and protein G purification steps. Aliquots of each step were analyzed on 10% Tris-Glycine gel-SDS. Proteins (6.5 µg) were loaded in each well and visualized with Sypro Ruby. 1= Commercial polyclonal IgG (control); 2= SD serum pre-Melon purification; 3= SD post-Melon purification; 4= SD elution 1 from protein G column; 5= SD elution 2 from protein G column; 6= CR serum pre-Melon purification; 7= CR post-Melon purification; 8= CR elution 1 from protein G column; 9= CR elution 2 from protein G column; 10= Commercial polyclonal IgG (control), MW 150 kDa. (**b**) and (**c**) SELEX progress was evaluated for SD and CR SELEX. The cycles of PCR required to amplify enough material (80 pmols) to advance to the next round of SELEX were recorded and used to compare SELEX rounds to evaluate progress. (**b**) At SD SELEX round 7 there is a significant drop in the amount of PCR cycles required (8 PCR cycles) as compared to previous SELEX rounds. (**c**) At CR SELEX round 6 there is significant drop in the PCR cycles required (9 PCR cycles) to amplify enough material as compared to previous SELEX rounds. In both cases this indicates enrichment of sequences. (**d**) Two family of sequences were isolated during SD SELEX. Only one group (aptS) bound to SD in serum. 37% of the 19 cloned sequences were aptS. (**e**) 5 family of sequences were isolated during CR SELEX. Only one group (aptC) bound to CR in serum. 10% of the cloned sequences were aptC.
