# Supplementary material for: Personalized immunoglobulin aptamers for detection of multiple myeloma minimal residual disease in serum
Source: Commun Biol. 2020 Dec 17;3:781. doi: 10.1038/s42003-020-01515-x (PMC7747622; doi:10.1038/s42003-020-01515-x)
Supplement: Supplementary file 3 — Description of Supplementary Files [file 42003_2020_1515_MOESM3_ESM.pdf]

Description of Additional Supplementary Files

Title: Supplementary Data 1.
Description: Raw data of all graphs and supplementary figures.
